# Chemical Diagrams, &c.

**Published:** 1834-01-01

**Authors:** 


*Chemical Diagrams, &c.* By ALEXANDER LEE, A.M., Surgeon, Editor and Translator of Celsus.—London, 1833. 12mo. pp. 182.

MR. LEE says very gravely, in the preface, "Let it not be supposed that it is intended to supersede the valuable works of Drs. Henry and Turner; such an idea was never entertained by me. Its proper use is, first, in the lecture-room, to enable the pupil more readily to understand the changes which the lecturer is describing, either verbally or by diagram." Mr. Lee might have saved himself the trouble of warning his readers not to mistake his intentions; for no person can fall into so grievous an error as to prefer his book to either Henry or Turner. Our opinion in respect to "its use in the lecture-room" is very different to that of the author; for, as all the lectures on chemistry that we have heard are furnished with much clearer diagrams than those produced by Mr. Lee, and, as we presume, this is also the case with those whom we have not heard lecture, the work will rarely be put to its first use.

The following passage is not correct:

"30. Nitrate of ammonia. The salt here employed is compounded of nitric acid and ammonia, and is destitute of water; nitric acid is compounded of 5 of oxygen and 1 of azote; ammonia is compounded of 3 hydrogen and 1 azote; both the nitric acid and the ammonia suffer complete decomposition, 3 atoms of hydrogen combine with 3 of oxygen to form 3 of water, the remaining 2 of oxygen then combine with 2 of azote resulting from the decomposition of both acid and base, to form nitrous oxide, which may be collected in its gaseous state; water and nitrous oxide gas are therefore the sole products of the decomposition of this salt.

"Nitrate of Ammonia 1 = 71.

| Nitric Acid 1 = 54 | | | Ammonia 1 = 17 | |
|---|---|---|---|---|
| Azote 1 = 14 | Oxygen 2 = 16 | Oxygen 3 = 24 | Hydrogen 3 = 3 | Azote 1 = 14 |
| | | Water 3 = 27 | | |

Protoxide of Azote, or Nitrous Oxide, 22 × 2 = 44."

If Mr. Lee had read either Turner or Henry with sufficient attention, (to say nothing of examining the salt himself,) he would not surely have stated that crystallized nitrate of ammonia contains no water. Any pupil who will take the trouble to refer to Turner, will find a much clearer account of the decomposition of this salt in procuring nitrous oxide than Mr. Lee has given; for protoxide of azote, which is formed and given off in a gaseous state, is placed at the bottom of Mr. Lee's diagram, while the nitrate of ammonia, which undergoes decomposition, and gives rise to the new compound, is placed at the top: this is certainly reversing the order of diagram-making, but it is by no means a solitary instance of Mr. Lee's perverting the order of things.

The work is principally a compilation from those books which are, or ought to be, in the hands of all medical and chemical students; and therefore we leave it to them to judge how far they may profit by it. Some of the tables, however, are certainly useful, and the form, as well as the price of the book, will make them accessible.

